# Experimental and virtual testing of bone-implant systems equipped with the AO Fracture Monitor with regard to interfragmentary movement

**DOI:** 10.3389/fbioe.2024.1370837

**Published:** 2024-03-08

**Authors:** Kerstin Wickert, Michael Roland, Annchristin Andres, Stefan Diebels, Bergita Ganse, Dorothea Kerner, Felix Frenzel, Thomas Tschernig, Manuela Ernst, Markus Windolf, Max Müller, Tim Pohlemann, Marcel Orth

**Affiliations:** ^1^ Applied Mechanics, Saarland University, Saarbrücken, Germany; ^2^ Werner Siemens Endowed Chair of Innovative Implant Development (Fracture Healing), Saarland University, Homburg, Germany; ^3^ Clinic of Diagnostic and Interventional Radiology, Saarland University Hospital, Homburg, Germany; ^4^ Institute of Anatomy and Cell Biology, Saarland University, Homburg, Germany; ^5^ AO Research Institute Davos (ARI), Davos, Switzerland; ^6^ Department of Trauma, Hand and Reconstruction Surgery, Saarland University Hospital, Homburg, Germany

**Keywords:** experimental biomechanics, biomechanical simulation, bone healing, orthopedic trauma surgery, osteosynthesis, smart implant, patient monitoring

## Abstract

**Introduction:** The management of fractured bones is a key domain within orthopedic trauma surgery, with the prevention of delayed healing and non-unions forming a core challenge. This study evaluates the efficacy of the AO Fracture Monitor in conjunction with biomechanical simulations to better understand the local mechanics of fracture gaps, which is crucial for comprehending mechanotransduction, a key factor in bone healing. Through a series of experiments and corresponding simulations, the study tests four hypotheses to determine the relationship between physical measurements and the predictive power of biomechanical models.

**Methods:** Employing the AO Fracture Monitor and Digital Image Correlation techniques, the study demonstrates a significant correlation between the surface strain of implants and interfragmentary movements. This provides a foundation for utilizing one-dimensional AO Fracture Monitor measurements to predict three-dimensional fracture behavior, thereby linking mechanical loading with fracture gap dynamics. Moreover, the research establishes that finite element simulations of bone-implant systems can be effectively validated using experimental data, underpinning the accuracy of simulations in replicating physical behaviors.

**Results and Discussion:** The findings endorse the combined use of monitoring technologies and simulations to infer the local mechanical conditions at the fracture site, offering a potential leap in personalized therapy for bone healing. Clinically, this approach can enhance treatment outcomes by refining the assessment precision in trauma trials, fostering the early detection of healing disturbances, and guiding improvements in future implant design. Ultimately, this study paves the way for more sophisticated patient monitoring and tailored interventions, promising to elevate the standard of care in orthopedic trauma surgery.

## 1 Introduction

The treatment of tibial and fibular fractures has seen significant experimental and clinical progress in recent years. However, the rates of delayed bone healing and non-union remain high, posing a substantial challenge to healthcare systems globally (Zura et al., 2016; Dailey et al., 2018). Fractures are a common occurrence in any society, affecting individuals regardless of age, social, or societal status. The majority of these fractures require surgical intervention for stabilization, a practice that has dramatically improved healing outcomes and functional restoration over the past 6 decades (Tzioupis and Giannoudis, 2007; Calori and Giannoudis, 2011).

Despite these advancements, complications such as infection, delayed healing, and non-union can frequently compromise the expected results, leading to increased treatment time, numbers of surgical interventions, and healthcare costs (Augat et al., 2005). The World Health Organization (WHO) identifies trauma as a “major healthcare epidemic,” contributing to 16% of the global disease burden. This is due, in part, to the high complication rates up to 10% (Fong et al., 2013) associated with fractures, particularly those of the tibia at the diaphyseal segment (Harris and Lyons, 2005), which are among the most common lower extremity fractures generating high treatment costs in case of non-unions (Dahabreh et al., 2007; Antonova et al., 2013).

The stability of the osteosynthesis plays a crucial role in the healing process. It directly influences interfragmentary movement (IFM), the relative motion between bone fragments (Claes, 2017). Understanding the mechanisms underlying the effects of IFM on fracture healing is of paramount importance in enhancing the treatment and management of fractures, ultimately leading to improved clinical outcomes. The AO Foundation has developed a medical device to record the relative loading of an implant that can be attached to a plate used to treat fractures (Windolf et al., 2022). Their goal is to enable clinical studies in human patients with continuous biomechanical recordings at the fracture site, as well as to improve clinical practice by offering live feedback on the healing progress.

The primary aim of the present study was to establish a link between the 1D continuous measurement signal from the AO Fracture Monitor and the 3D outcome of the corresponding biomechanical simulation regarding the local fracture mechanics within the bone-implant system. To investigate this, the following four hypotheses and methodological aims were formulated:

(1) Digital Image Correlation (DIC) outcomes and the IFM correlate in experiments on cadaveric bone-implant systems conducted in a specialized testing device.
(2) From the data recorded by the AO Fracture Monitor, it is possible to predict the DIC results for the IFM, connecting the implant loading with the fracture gap behavior.
(3) The quality of simulation results can be assessed using the IFM DIC data.
(4) A linear model can be determined for conversion of AO Fracture Monitor data to simulated IFM, transitioning from 1D to 3D.

## 2 Materials and methods

### 2.1 Specimen preparation, surgical procedure and imaging

**FIGURE 1:**
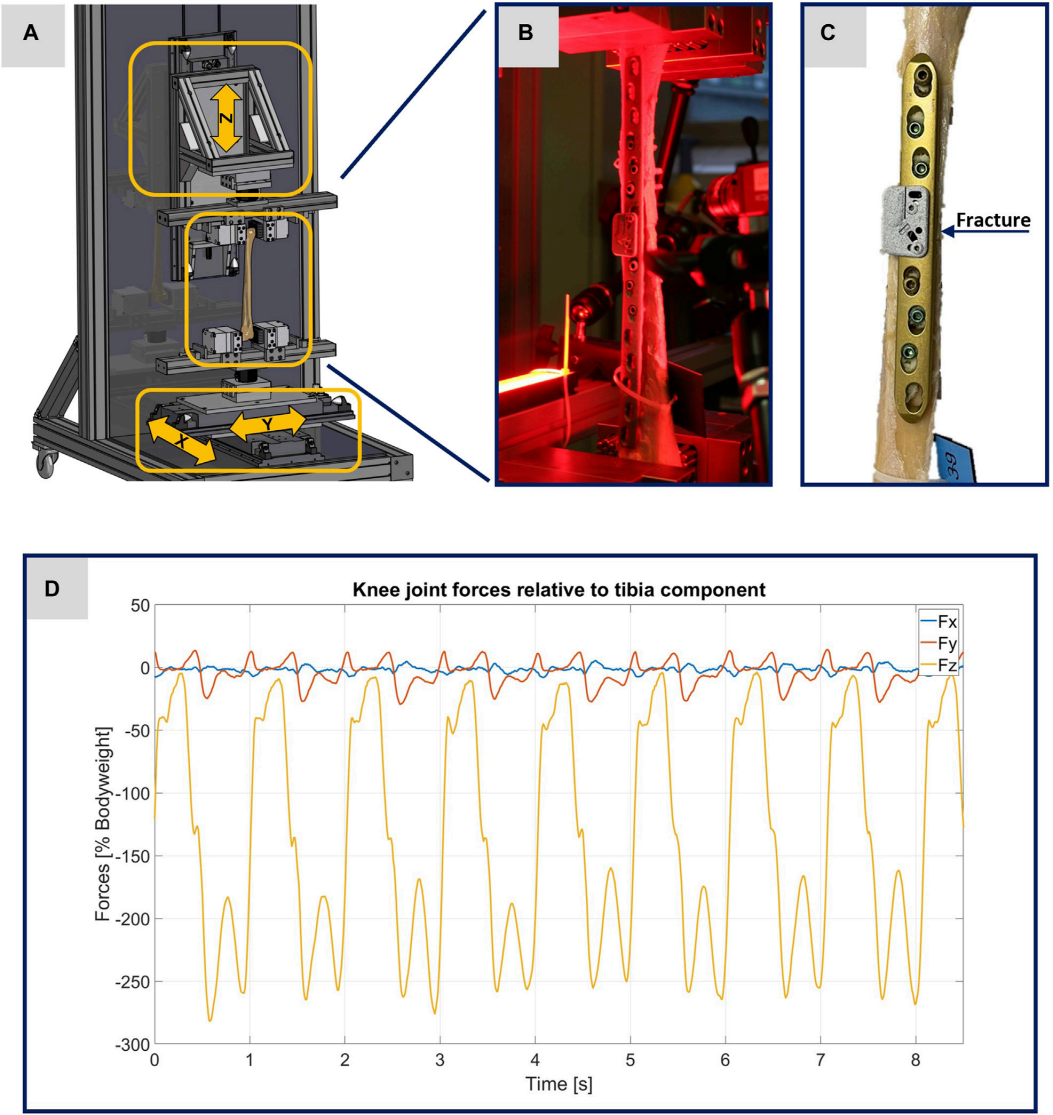
Illustration of the experimental setup. **(A)** Computer-aided design representation of the testing device. The three arrows show the direction of movement of the linear modules. **(B)** Photo of a specimen clamped in the testing device before the start of an experiment. **(C)** Photo of the specimen 2139_1_ as exemplary image of the treated tibiae. **(D)** Plot of the original data from the OrtholoadTM database representing the scalable input parameters for the testing device.

The cadaveric specimens (*n* = 3) came from body donations and were fixed with a nitrite pickling salt solution (Janczyk et al., 2011). The specimens were obtained from a male donor (left and right tibia), age of death 56 years, and from a female donor (left tibia), age of death 81 years. The cadaveric tibiae were used to induce osteotomies in the diaphyseal area of the bones. The osteotomies were performed using a regular clinical saw with a thickness of 0.9 mm (Colibri II, DePuy Synthes, Norderstedt, Germany) and were shaped as transverse fractures equivalent to a fracture type 42-A3 according to the AO classification (Müller et al., 1990). The osteotomized bones were then anatomically reduced and stabilized by limited contact-dynamic compression plates (LC-DCP) 4.5/5.0 mm (DePuy Synthes). The resulting gap size was measured between 0.0–0.9 mm in the area of the osteotomy. During osteosynthesis, the plates were first fixated to the bones using cortical screws to induce the dynamic compression (DC) effect according to AO principles (Buckley et al., 2018). Further plate fixations were performed by locking screws. The prepared specimen from the female donor is exemplified in Figure 1C. The three specimens are named as follows throughout this manuscript: 2131_1_ (male donor, left tibia), 2131_2_ (male donor, right tibia) and 2139_1_ (female donor, left tibia).

The geometric models for the biomechanical FE simulations are based on computed tomography (CT) scans conducted from each of the specimens, including a six-rod bone density calibration phantom (QRM-BDC/6, QRM GmbH Moehrendorf, Germany). All bone-implant systems were scanned with a commercially available CT scanner (Somatom Definition AS64, Siemens Healthineers, Erlangen, Germany). The bone-implant systems were placed longitudinally on the CT table with the phantom being positioned under it. Spiral CT acquisitions were performed using the following specifications: tube voltage: 120 kVp, tube current: 95 mA, collimation of 64 × 0.6 mm and a pitch factor of 0.8. Further postprocessing included reconstruction of axial slices with a slice thickness of 3 mm (increment of 2 mm) and 0.6 mm (increment of 0.4 mm) applying a bone reconstruction kernel (B70s) and a soft tissue reconstruction kernel (B31s) respectively.

The AO Fracture Monitor is an active implantable medical device for continuous monitoring of bone fracture healing (Ernst et al., 2021a; Ernst et al., 2021b; Windolf et al., 2022). The AO Fracture Monitor will be attached to conventional bone plates (DePuy Synthes) via two adjacent empty screw holes on the fracture bridging plate segment. A strain gauge measures relative loading of the bone plate. The data are accessed via a Bluetooth connection with the accompanying Smartphone application at a sampling rate of 10 Hz. In normal use, the data are internally processed into relevant metrics and stored on the AO Fracture Monitor. Recorded data are transmitted automatically via Bluetooth to the patient’s smartphone once a day and accessible to the physician via a web application. During the experiments presented here, the data were also recorded at 10 Hz, but were not processed internally, but transferred directly via Bluetooth and smartphone for further downstream analysis.

Before performing the experiments, the AO Fracture Monitor was applied right above the fracture in each of the three specimens. For this purpose, two inserts were attached to the implant with a torque of 4 Nm. The AO Fracture Monitor was fixed in each case to the inserts with two screws (tightening torque 1.5 Nm). Typical placements of the AO Fracture Monitor are illustrated in Figures 1B, C.

For the evaluation of the experiments via DIC, it was necessary to apply a speckle pattern to the specimens. A white spray paint was used to create an even surface. Then a black spray paint was used to create an artificial speckle pattern. To achieve better results and reduce disturbing influences, red light filters were used for the cameras and the specimens were illuminated with red light, cf. Figure 1B.

### 2.2 Mechanical testing device and testing protocol

All experiments were performed using a specially designed testing device. The operational scope of this testing device is to precisely simulate different loading scenarios of treated human tibiae during walking, with the goal to enhance the comprehension of the interplay between bone and implant. To implement the requirements addressed here in the testing device, forces were applied longitudinally (in the *z*-direction) and transversely (in the x- and y-directions) to the bone axis using linear modules, as illustrated in Figure 1A. The maximum possible force applied along the bone axis is limited to 5 kN and the maximum possible stroke is set to 235 mm. Transverse force application is accomplished using two linear modules, responsible for bone displacement in the x- and y-directions, with maximum possible forces limited to 2 kN and maximum possible strokes to 60 mm. The specimens were held in place using an individual flexible mold clamping system, consisting of multiple pins that conformed perfectly to the complex shape of the bone (Silver Clamp X-Clamp, MATRIX GmbH, Ostfildern, Germany). The tibial plateau was secured in the lower clamp, pointed upwards, while the distal epiphysis was clamped to the upper clamp. This type of clamping facilitates the force application with regard to the selected linear modules and their control and regulation technology.

To guarantee the authenticity of the loading scenarios, input parameters from Orthoload™ were used, specifically the dataset from subject k8l_191,211_1_107p (male participant with a body weight of 755.0 N), which exemplifies knee forces during walking on a treadmill for eight steps (Orthoload database, 2023). Each specimen was subjected to two tests, one at full weight bearing representing a normal step forward, and another test at 50% of the forces representing a partial weight bearing scenario. Figure 1D shows the original data from the Orthoload database, which, scaled according to the specifications, were used as input parameters for the testing device.

Throughout the experiments, the testing device mapped the forces from the eight steps of the input file onto the donor bones with a machine velocity of also 10 Hz. During the experiments, a six-axis force sensor (K6D80, ME-Meßsysteme GmbH, Henningsdorf, Germany) was affixed to each mold clamping system for real-time monitoring of the forces exerted. This setup verified the application of force data from the scaled Orthoload™ file at a 10 Hz frequency.

For further experimental analysis, the recording was executed using a two-camera system calibrated for subsequent three-dimensional DIC, with real-time synchronization ensured by a trigger box (DAQhw-Triggereinheit, LIMESS Messtechnik und Software GmbH, Krefeld, Germany) linked to the CompactRIO (C-RIO, National Instruments, Austin, United States). This setup captured the dynamics of the implant surface, the AO Fracture Monitor, and the fracture gap, facilitating a comprehensive optical evaluation. Strain comparisons on the implant and AO Fracture Monitor surfaces, as well as the IFM, were analyzed using the ISTRA 4D 4.4.4.694 software (Dantec Dynamics, Skovlunde, Denmark). The standard deviation in DIC is 1 per thousand (0.001).

### 2.3 Simulation workflow

The geometric models used in this study are based on CT imaging. The dicom image stacks of the treated tibiae including the six-rod bone density calibration phantom were segmented into different masks (bone, implant, fracture, single rods) with an adaptive threshold procedure, supplemented by a morphological close filter with isotropic values, an island removal, a cavity fill, and a mask smoothing with a recursive Gaussian filter with anisotropic values resulting in a high segmentation quality without detectable problems. The segmentation procedure as well as the following meshing strategy were performed in the image processing and model generation software Simpleware™ ScanIP (Synopsys, Mountain View, CA, United States). The material parameters of the osteosynthesis (Young’s Modulus: 108,000 MPa, Poisson ratio: 0.375, standard values for medical titanium alloys) (Imam and Fraker, 1996) and the fracture gap (Young’s Modulus: 3 MPa, Poisson ratio: 0.4, values for initial connective tissue in fracture healing) (Claes and Heigele, 1999) were chosen from literature as homogeneous parameters.

**FIGURE 2:**
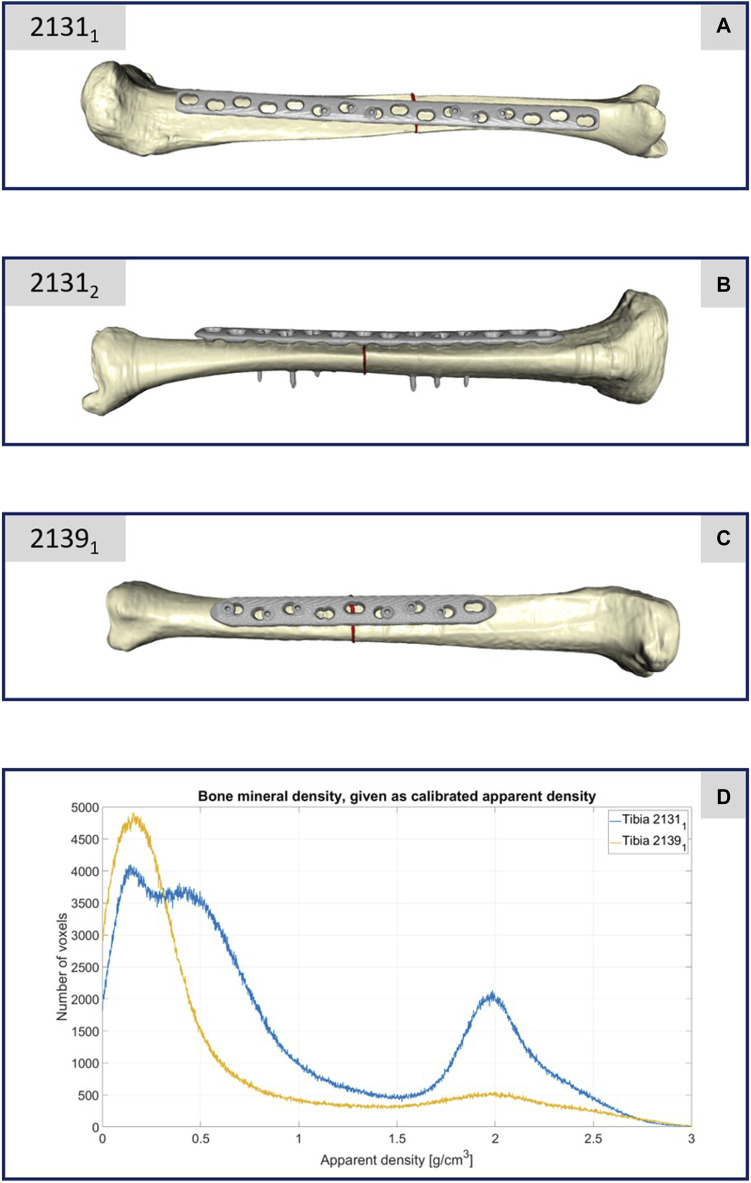
**(A–C)** Illustration of the computational models of the three different treated tibiae. **(D)** Plot of the bone mineral density of the two specimens 2131_1_ and 2139_1_ with respect to the apparent density and the corresponding number of CT voxels.

The material properties of the three tibiae were carefully characterized and calibrated with respect to the data given by the calibration phantom. A four-step procedure was performed to transfer the grayscale values in Hounsfield units (HU) from the CT images to local bone properties. (1) A densitometric relationship defining a mapping to convert raw CT attenuation to bone mineral density (BMD) values was defined for each tibia. Therefore, histograms were generated for each rod of the calibration phantom, reflecting the corresponding grayscale values relative to their voxel count. These histogram curves were then smoothed using a robust local regression method. Curve maxima were used as calibration points for a least square fit, which defines the required mapping of CT data in HU to equivalent mineral density values. (2) In the second step, the ash density values were calculated from the equivalent mineral density values via the relationship for hydroxyapatite phantoms from (Eberle et al., 2013). (3) These values were then used to calculate the apparent density values with the conversion formula given in (Edwards et al., 2013). (4) The last step was the mapping to the material parameters via the density-modulus relationship introduced in (Rho et al., 1995). In accordance with the work of (Cattaneo et al., 2001), a total of 25 different bone material cards were defined and stored in the computational meshes. All these computations were performed in Matlab (Matlab 2021b; MathWorks, MA United States). After the material assignment, the areas close to the joints (foot and knee) were also marked in the ScanIP software during the meshing in order to be able to apply the boundary conditions correctly in the simulation environment and thus be able to represent the clamping in the testing device realistically in the simulations. Figures 2A–C shows the generated computational models of the three specimens.

For the meshing strategy, quadratic tetrahedral finite elements of C3D10 type, characterized by straight edges, were chosen and the meshes were adaptively refined with respect to the implants and fracture areas resulting in numbers of mesh cells given in Table 1. All biomechanical FE simulations were performed in the finite element analysis and computer-aided engineering software suite Abaqus (Dassault Systemes, Velizy-Villacoublay, France). Once the three models were imported into Abaqus, the initial step was to align the bone-implant systems properly. The reference for alignment was established using the coordinate system provided by Orthoload™. To mimic the experimental conditions, the second forward step was simulated with careful consideration of the boundary conditions. The boundary conditions were implemented to accurately replicate the experimental setup. Specifically, the x- and y-components of the knee joint force were applied to the tibial plateau, while the z-component remained fixed. At the distal end, the z-component of the knee joint force was applied, while the components in the x- and y-directions were constrained. The simulation proceeded by systematically iterating through all the force values until all the force components of the forward step were effectively accounted for and simulated. This approach ensured a comprehensive analysis of the biomechanical response during the simulated step forward. By meticulously replicating the experimental conditions and considering the appropriate boundary conditions, the simulations provided a valuable opportunity to gain insights into the behavior and response of the bone-implant systems under different loading conditions representing full and partial weight bearing.

**TABLE 1:** Number of mesh cells (quadratic tetrahedral elements of Abaqus type C3D10 with straight edges) per segmented mask.

		Specimen		
		2131_1_	2131_2_	2139_1_
Segmented mask	Implant	179,125	201,067	149,101
	Fracture	56,895	87,649	71,855
	Bone, all material cards	964,391	1,088,442	657,814

### 2.4 Statistical analyses and data processing

For hypothesis 1, we conducted Pearson correlation analyses to assess the relationship between the DIC measurements of the IFM and the implant across all eight experimental steps. Furthermore, we derived mean curves from the collective dataset and adjust these curves at the baseline of zero. Subsequently, we employed linear regression analysis to examine the predictive relationship, treating the implant data as independent variable and the IFM data as the dependent variable.

For hypothesis 2, we conducted a comparative analysis of the data from the AO Fracture Monitor and the corresponding IFM data from the DIC evaluation. The raw data from the eight steps were initially processed to compute their mean curves. This entailed averaging the values across all eight steps at each point in time, providing a representative trend for the collective behavior observed during the experiments. To ensure a common basis for comparison, the mean curves were interpolated across a standardized set of time points, facilitating an accurate correlation and regression analysis between the datasets. The interpolated mean curves were then adjusted to the origin to negate any initial offset and align the starting points of both datasets. Linear regression models were employed to predict the IFM values based on the AO Fracture Monitor data, utilizing the adjusted mean curves. The model’s performances were quantified using the mean squared error (MSE) and the coefficient of determination (*R* ^2^), with a high *R* ^2^ value indicating a strong predictive capability of the model.

**FIGURE 3:**
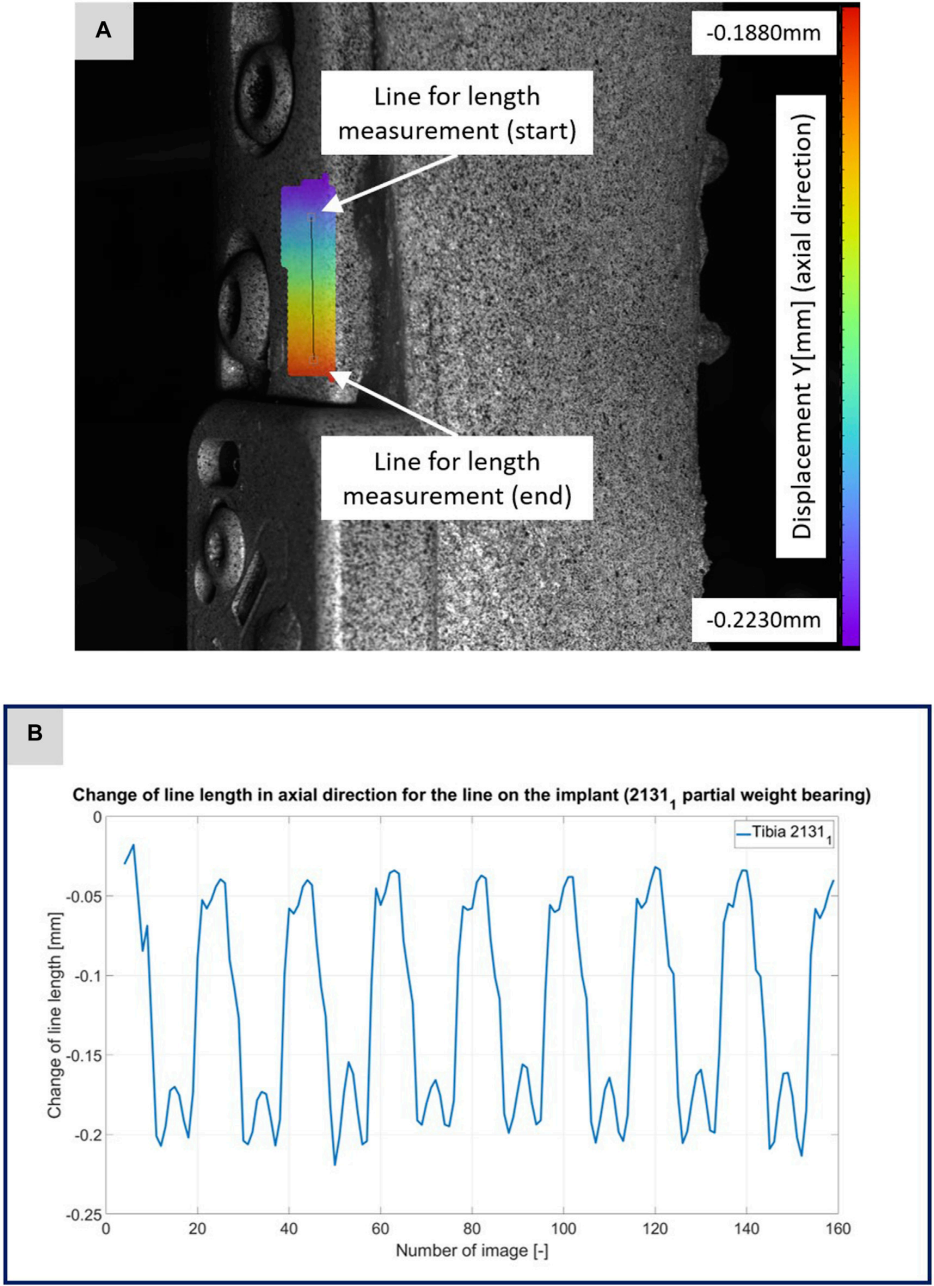
**(A)** Illustration of the DIC analysis for a line virtually placed on the implant for specimen 2131_1_. The color bar shows the displacement in axial direction with a minimum value of −0.2230 mm and a maximum value of −0.1880 mm. **(B)** The plotted curve shows the change of the line length over the experiment.

**FIGURE 4:**
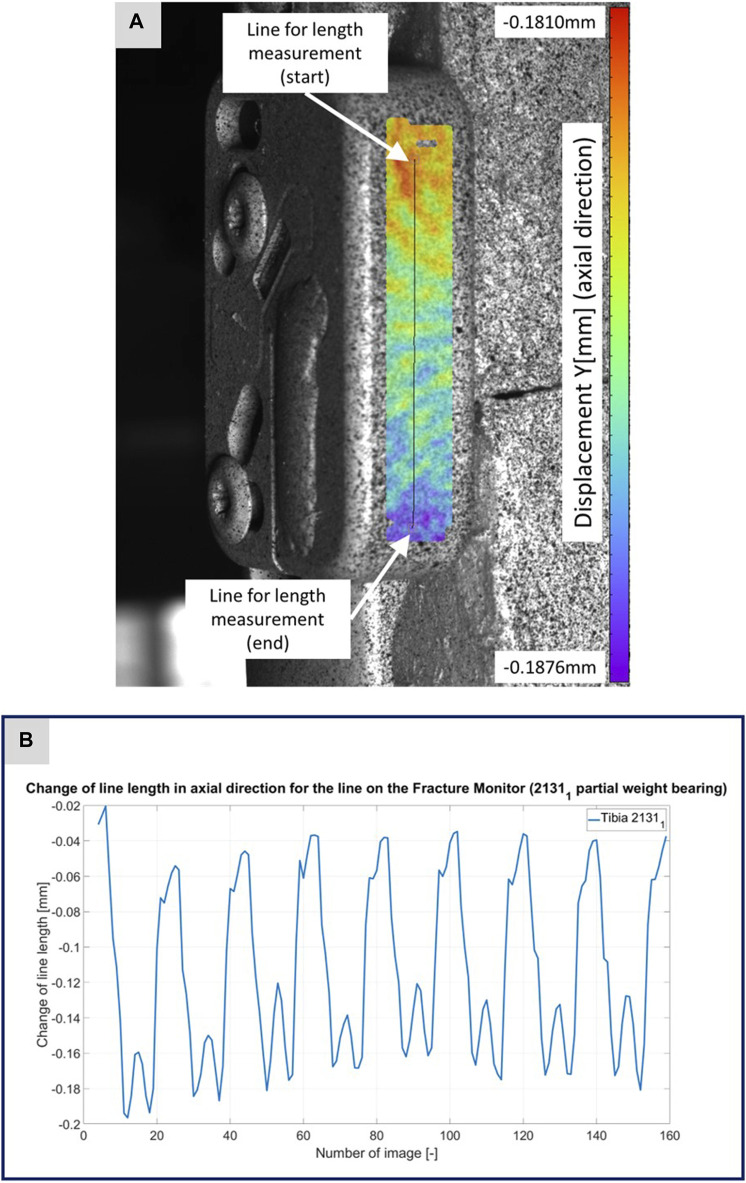
**(A)** Illustration of the DIC analysis for a line virtually placed on the Fracture Monitor for specimen 2131_1_. The color bar shows the displacement in axial direction with a minimum value of −0.1876 mm and a maximum value of 0.1810 mm. **(B)** The plotted curve shows the change of the line length over the experiment.

**FIGURE 5:**
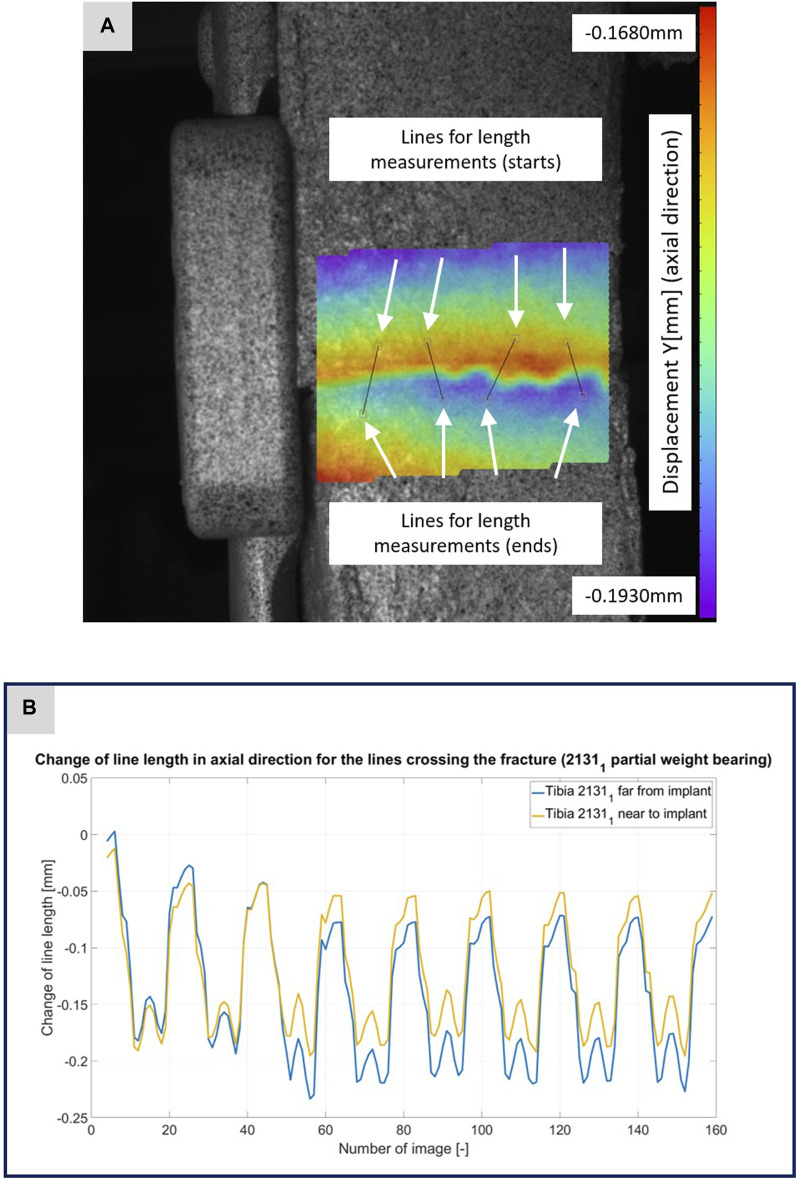
**(A)** Illustration of the DIC analysis for four lines virtually placed across the fracture gap for specimen 2131_1_. The color bar shows the displacement in axial direction with a minimum value of −0.1930 mm and a maximum value of −0.1680 mm. **(B)** The plotted curves show the change of the line lengths over the experiment for the line near to implant and the line far from implant.

In order to investigate hypothesis 3 and thus to compare the simulation results with the experiments conducted, the following procedure was executed: (1) the displacement data, computed using the DIC analysis software ISTRA 4D, were saved alongside the initial or base lengths of the evaluation lines (refer to Figures 3–5). (2) Both the image data and the DIC results derived from them were calibrated in a manner similar to the AO Fracture Monitor results, considering the beginning of the experiment. (3) The length changes at each time point were computed and compared to the base lengths to ascertain the strains throughout the experiment’s duration. (4) Analogous to the lines chosen in the DIC analysis, points were selected on the FE mesh surfaces, and both their coordinates and corresponding displacements were recorded. (5) The saved coordinates were utilized to establish the base lengths of the lines on the FE meshes. The associated displacements were then employed to calculate the changes in length and subsequently the strains. This process provided IFM curves from the simulations, which served as a virtual equivalent to the experimentally obtained IFM curves.

For the comparison of results, mean value curves were calculated from the experimental IFM curves over all eight steps and these were compared with the virtual IFM curves obtained from the simulation. For this purpose, the experimental IFM curves were interpolated to the same time points, since the simulations were performed with a slightly higher time resolution of the forward step than the DIC evaluation. Thereafter, the Euclidean distance and the MSE between the simulated and experimental curves were calculated for all experiments, as well as the Pearson correlation coefficient between the data of the curves.

To explore hypothesis 4, which postulates a correlation of the 1D signal from the AO Fracture Monitor and the simulated 3D local micro-mechanics within the fracture gap, a systematic approach was undertaken. The methodology entailed the computation of the stress and strain tensors at each integration point followed by averaging over every mesh cell. Subsequent to this, pertinent strain metrics such as octahedral shear strain and hydrostatic strain were derived, which are essential for the following 3D evaluation of the healing window, cf. (Claes and Heigele, 1999; Shefelbine et al., 2005). Furthermore, to facilitate a comparative analysis with the AO Fracture monitor’s 1D output, the strain energy density was also computed. The strain energy density describes the amount of strain energy stored in a material per unit volume due to deformation and is a comparative measure offering benefits for analyzing bone-implant systems. It simplifies complex data from 3D simulations into a scalar value without losing crucial information and therefore acts as a unified metric that effectively captures multi-axial strain states. Statistical values, including sum, mean, median, maximum, interquartile range (IQR) and 90th percentile (90th PRC) of the strain energy density, were computed across the fracture gap. These measures were then cross-correlated with the AO Fracture Monitor’s mean value curves from the corresponding experiments. In addition, linear regressions were computed to predict the statistical values of the strain energy density from the AO Fracture Monitor mean curves.

## 3 Results

### 3.1 Experimental results

Each specimen was subjected to the two described loading scenarios, representing the forces for a step forward with full weight bearing and with 50 percent of the forces describing a step forward with partial weight bearing. During the experiment under full weight bearing, one of the three specimens (specimen 2139_1_) failed and fractured inside the clamping system.

The CT scan data of this specimen confirmed its lower bone mineral density, as depicted in Figure 2D. This figure compares the bone mineral density as apparent density over the number of voxels between specimens 2139_1_ and 2131_1_, highlighting considerably reduced density in 2139_1_, particularly in areas typically dense in cortical bone. This discrepancy is attributed to the older age and different sex of the donors, with 2139_1_ being from an elderly female and 2131_1_ from a middle-aged male. Given the negligible difference in density between the male donor’s limbs, only one was depicted in Figure 2D. Thus, specimen 2139_1_ underwent testing only with a decreased load, representing partial weight bearing.

Figures 3–5 illustrate the DIC analyses process. Figure 3 displays the axial displacement for a small section of the implant surface. The timing of this snapshot corresponds to the moment when the axial force specification reached its maximum during the second step in the input data. In addition to the displacement field, the temporal change in line length throughout the entire experiment was plotted as a curve (Figure 3B). This curve mirrors the progression of the force specification (input parameter of the testing device) as depicted in Figure 1D. The typical double-peak structure with its two maxima is visible in the curve. Figure 4 displays the axial displacement field on the lateral surface of the AO Fracture Monitor at the same time point. Analogous to Figure 3, the plot of the line length change over the entire course of the experiment also shows the double-peak curve for the eight steps from the input file. In contrast to the length change on the implant surface, there are larger differences in the data of the individual steps here. This could be due to the fact that the AO Fracture Monitor, as described, was screwed onto the implant, thereby creating a connection with a small amount of play between the force application and the measurement surface. Figure 5 presents the result of the length change of the same specimen 2131_1_ at the same time point as Figures 3, 4. The two plotted lines represent the length change near the implant and the length change of the line furthest from the implant, respectively. Again, the double-peak pattern is evident, analogous to the other results. However, it can be observed that the line length at the end of the experiment did not return as far towards the initial length as in the case of the implant. This could be due to the fact that the fracture gap was empty and not filled with a suitable material.

For hypothesis 1, Table 2 presents Pearson correlation coefficients that revealed a significant linear relationship at the 0.01 level between the DIC results for the implant and the IFM for all specimens. The consistently high coefficients across all steps, particularly under partial weight bearing conditions, indicate a strong adherence to the force application protocol. Specimen 2139_1_, tested only under a reduced load, showed significant correlation values exceeding 0.89, underscoring the reliability of the experimental design.

**TABLE 2:** Correlation coefficients from a Pearson test for the DIC data evaluated for the implant and for the IFM. All correlations are significant at the 0.01 level.

Step	Specimen				
	2131_1_		2131_2_		2139_1_
	Full weight bearing	Partial weight bearing	Full weight bearing	Partial weight bearing	Partial weight bearing
1	0.9899	0.9989	0.9592	0.9989	0.9980
2	0.9839	0.9902	0.9348	0.8887	0.8986
3	0.9948	0.9505	0.8548	0.9993	0.9965
4	0.9845	0.9995	0.9243	0.9996	0.9965
5	0.9918	0.9991	0.8548	0.9995	0.9969
6	0.9911	0.9991	0.8719	0.9997	0.9967
7	0.9835	0.9995	0.8932	0.9997	0.9975
8	0.9871	0.9989	0.8608	0.9996	0.9963

**FIGURE 6:**
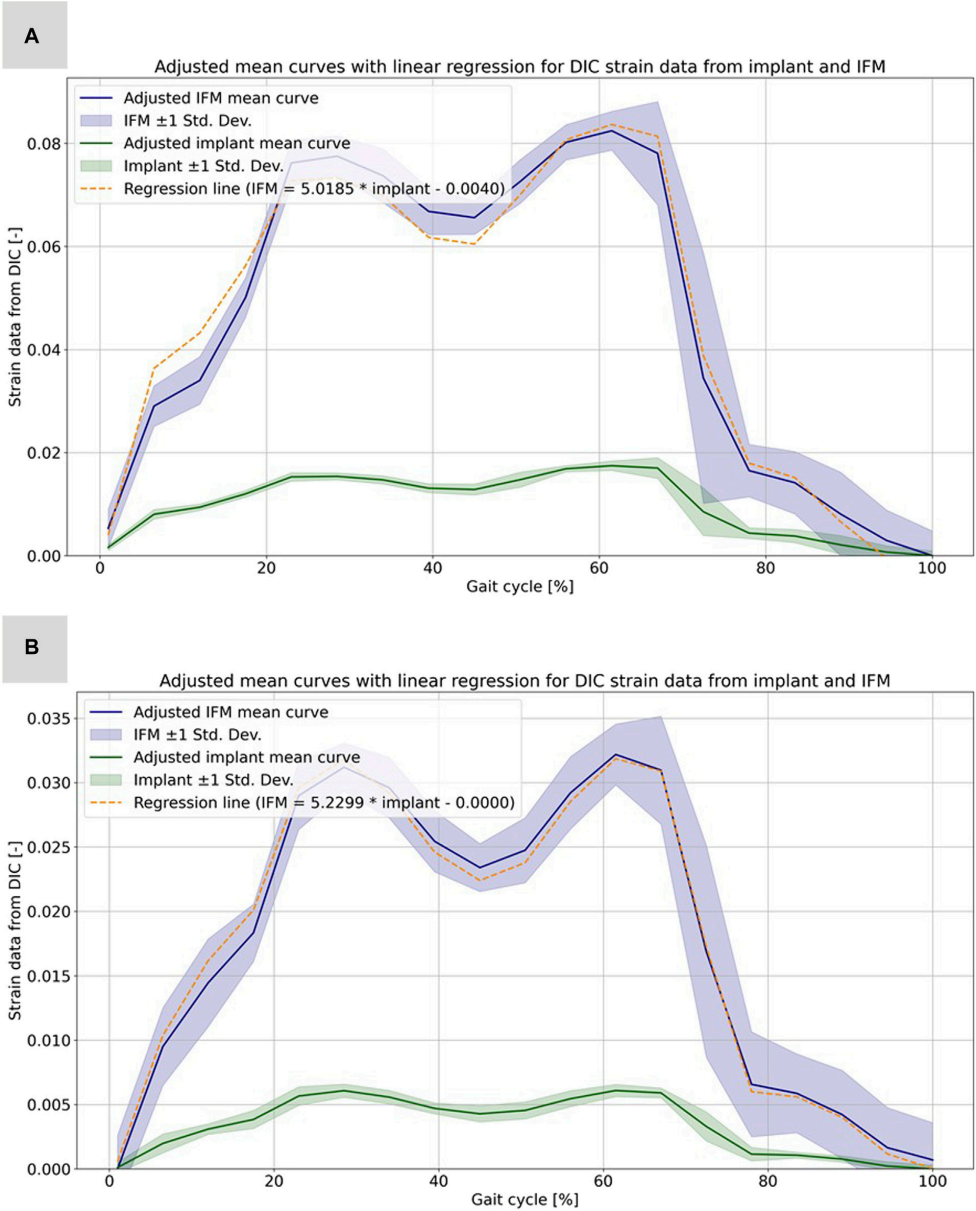
Illustration of the calculated strain data from the evaluation of the DIC data for the implant and the IFM for specimen 2131_1_. **(A)** Full weight bearing experiment: mean curves for the IFM and the implant data together with shaded error bars representing one standard deviation and the linear regression as dashed line. **(B)** Partial weight bearing: same curves over the full gait cycle.

Based on the high correlation coefficients, the linear regressions between the mean curves of the DIC results were computed for all five experiments. For this purpose, the mean curves were adjusted with respect to the origin, which is due to the slightly different setup weight of the testing device, which partially (clamping, holder, etc.) loads on the specimen. Figure 6 illustrates this procedure exemplarily for specimen 2131_1_ as the other experiments and specimens show very similar results. All linear regression functions are listed in Table 3 and show a strong linear regression for each specimen under full and partial weight bearing conditions. Notably, specimen 2139_1_ under partial weight bearing presented the highest correlation (*R* ^2^ = 0.9995), indicating an almost perfect linear relationship. These regressions provide a reliable model for predicting the IFM based on implant surface strain data received by DIC evaluation. The slopes of the regression lines vary only between 5.0185 and 5.6297 for the two specimens 2131_1_ and 2131_2_. This is expected, as both bones came from the same donor, were exposed to the same loading conditions and had similar fractures and treatments. Nonetheless, the substantial difference in the value of 2139_1_, which stands at 10.2688, precludes the possibility of making broad generalizations in this context.

**TABLE 3:** Functions of the linear regression (cf. Table 2) including the coefficient of determination *R* ^2^.

Specimen	Loading	Linear regression	*R* ^2^
2131_1_	Full weight bearing	IFM = 5.0185 * implant—0.0040	0.9799
	Partial weight bearing	IFM = 5.2299 * implant—0.0000	0.9949
2131_2_	Full weight bearing	IFM = 5.0689 * implant—0.0162	0.8504
	Partial weight bearing	IFM = 5.6297 * implant—0.0002	0.9984
21391	Partial weight bearing	IFM = 10.2688 * implant—0.0008	0.9995

The experiments conducted with the AO Fracture Monitor delivered seamless signal monitoring throughout the entire process. Utilizing the live mode feature, the data was recorded in real time and could be accessed and followed on a smartphone through Bluetooth technology. Furthermore, the datasets were transmitted to a cloud server with a time stamp after every measurement, processed and sent back for analysis. For hypothesis 2, the regression analysis revealed significant linear relationships between AO Fracture Monitor and IFM datasets, as evidenced by the high correlation coefficients and the close fit of the regression models to the observed data, cf. Table 4. The analysis under full weight bearing conditions for specimen 2131_1_ showed a correlation coefficient of 0.8673 with an MSE of 3.06e-05 and an *R* ^2^ of 0.9799. Under partial weight bearing, the correlation coefficient improved to 0.8954, with a slightly higher MSE of 3.27e-05 and an improved *R* ^2^ of 0.9949.

**TABLE 4:** Results of the linear regression analysis for the AO Fracture Monitor data (FM) and the corresponding experimental IFM data. The table shows the correlation coefficient between the datasets, the mean square error, the coefficient of determination *R* ^2^, and the linear regression to compute the IFM from the corresponding FM data for all five experiments.

Specimen	Loading	Correlation coefficient	MSE	*R* ^2^	Linear regression
2131_1_	Full weight bearing	0.8673	3.06e-05	0.9799	IFM = 3.025e-04 * FM + 0.01321
	Partial weight bearing	0.8954	3.27e-05	0.9949	IFM = 2.111e-04 * FM + 0.00452
2131_2_	Full weight bearing	0.9921	3.19e-05	0.8504	IFM = 1.468e-03 * FM—0.08425
	Partial weight bearing	0.9887	1.96e-05	0.9984	IFM = 9.476e-04 * FM + 0.01128
21391	Partial weight bearing	0.9901	1.14e-05	0.9995	IFM = 5.227e-04 * FM + 0.00803

**FIGURE 7:**
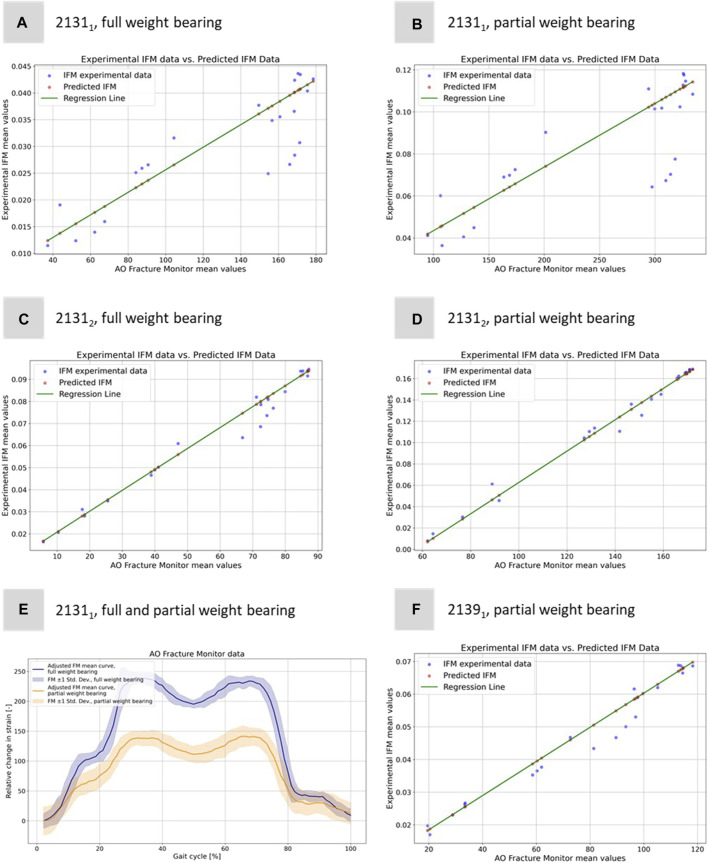
Illustration of the regression model to predict the experimental IFM data from the corresponding 1D AO Fracture Monitor data. Results for **(A)** specimen 2131_1_ under full weight bearing, **(B)** specimen 2131_1_ under partial weight bearing, **(C)** specimen 2131_2_ under full weight bearing, **(D)** specimen 2131_2_ under partial weight bearing, and **(F)** specimen 2139_1_ under only partial weight bearing. **(E)** Exemplary adjusted AO Fracture Monitor data for specimen 2131_1_ for full and partial weight bearing.

For Specimen 2131_2_, the full weight bearing condition yielded a very high correlation coefficient of 0.9921, an MSE of 3.19e-05, and a lower *R* ^2^ of 0.8504 compared to partial weight bearing, which demonstrated a correlation coefficient of 0.9887, an MSE of 1.96e-05, and a very high *R* ^2^ of 0.9984, indicating a near-perfect fit. Specimen 2139_1_, which was only evaluated under partial weight bearing, exhibited a correlation coefficient of 0.9901, with the lowest MSE in the dataset at 1.14e-05, and the highest *R* ^2^ value of 0.9995, denoting an almost exact predictive relationship in the regression model. Figure 7 shows all five regressions. The relatively high quality of the predictions is particularly evident in Figures 7C, D. However, the values of the straight-line slopes for samples 2131_1_ and 2131_2_ are not as similar as for the linear regressions for the different DIC evaluations.

The idea of computing these linear regressions was to link the measurements of the IFM from DIC and the AO Fracture Monitor to provide a better understanding of the IFM in clinical patients where additional data to the AO Fracture Monitor signal is neither available nor measurable. Figure 7E shows exemplarily the AO Fracture Monitor data for specimen 2131_1_ for full and partial weight bearing.

The curves were adjusted to the origin, as the AO Fracture Monitor only measures relative changes and the implant load is subject to slight variations at the start of the experiments due to the setup weight of the testing device, which partially (clamping, holder, etc.) also loads on the specimen. Figure 7E clearly reproduces double-peak stance-phase curve, cf. Figure 1D of the knee joint forces. In addition, the difference between full and partial weight bearing is visible and is around 1.79, i.e., slightly less than the expected factor of 2, but of the same order of magnitude as for specimen 2131_2_.

### 3.2 Biomechanical simulation results

**FIGURE 8:**
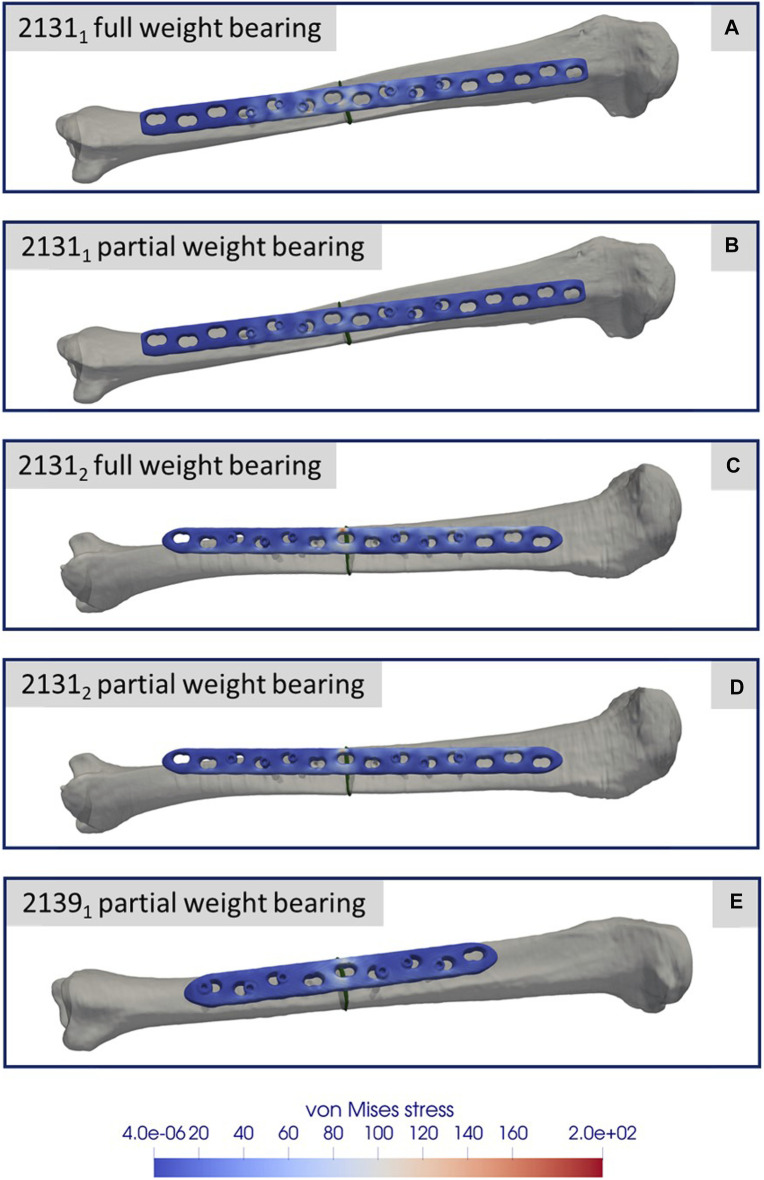
Simulation results for the three different specimens and the considered weight bearing cases **(A–E)**. All images show the von Mises stress distribution of the implant at the time point of the maximal axial force during a normal step forward.

Figures 8A–E depicts the simulation results at the moment of peak axial loading during the forward step. This figure specifically highlights the von Mises stress distribution on the implant in each case, since these key indicators have a high informative value with regard to a possible implant failure. Notably, even at the zenith of axial loading during full weight bearing and naturally during reduced partial weight bearing, the stress values peak at only 200 GPa. These peak values are localized above the fracture during full weight bearing and remain well below the stress thresholds that might induce failure.

**FIGURE 9:**
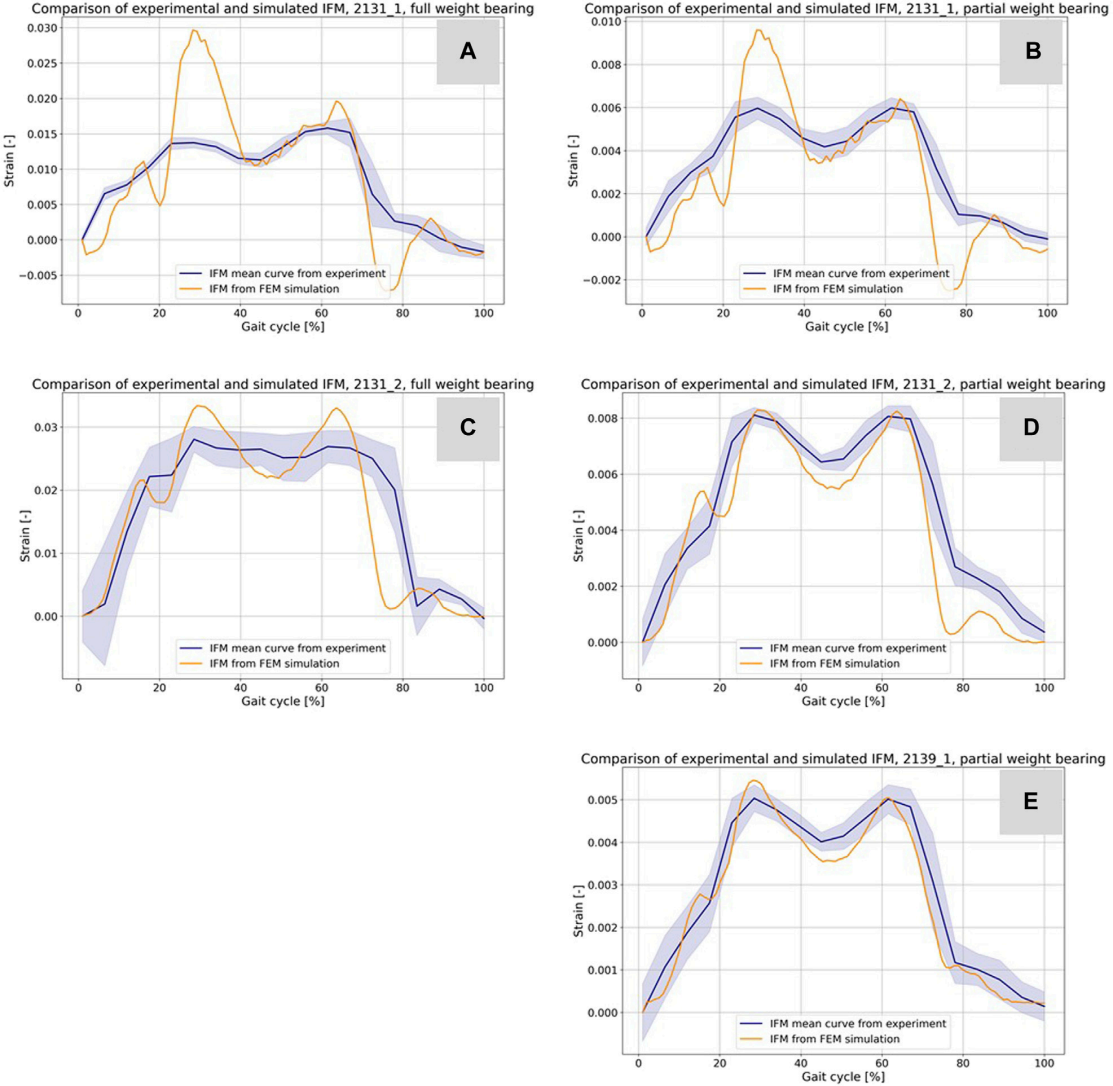
Comparison of the IFM from DIC of the experiments (cf. Figure 5) and the corresponding result based on the simulations **(A–E)**. The curves combined with a band representing the standard deviation show the experimental results and the orange curves show the counterpart from the simulations.

Figures 9A–E presents a comparison between the IFM derived from the DIC of the experiments (refer to Figure 5) and the results from the simulations. The blue curves depict the average IFM across all eight steps, with the band indicating the standard deviation from the experiments. In contrast, the orange curves represent the simulation results. Specifically, in Figures 9A, B for specimen 2131_1_, the simulation values exceeded those of the experiments. This discrepancy might arise from minor differences in the specimen’s orientation between the experiment and the simulation. While simulations ensure optimal alignment, clamping, and force transmission, experiments might introduce slight inaccuracies due to potential errors in these areas. However, the curves still closely resemble the experimental outcomes. For Figures 9C, D, concerning sample 2131_2_, the simulation closely mirrors the experimental results. Moreover, Figure 9E for sample 2139_1_ showcases an excellent match between experimental and simulation results.

**FIGURE 10:**
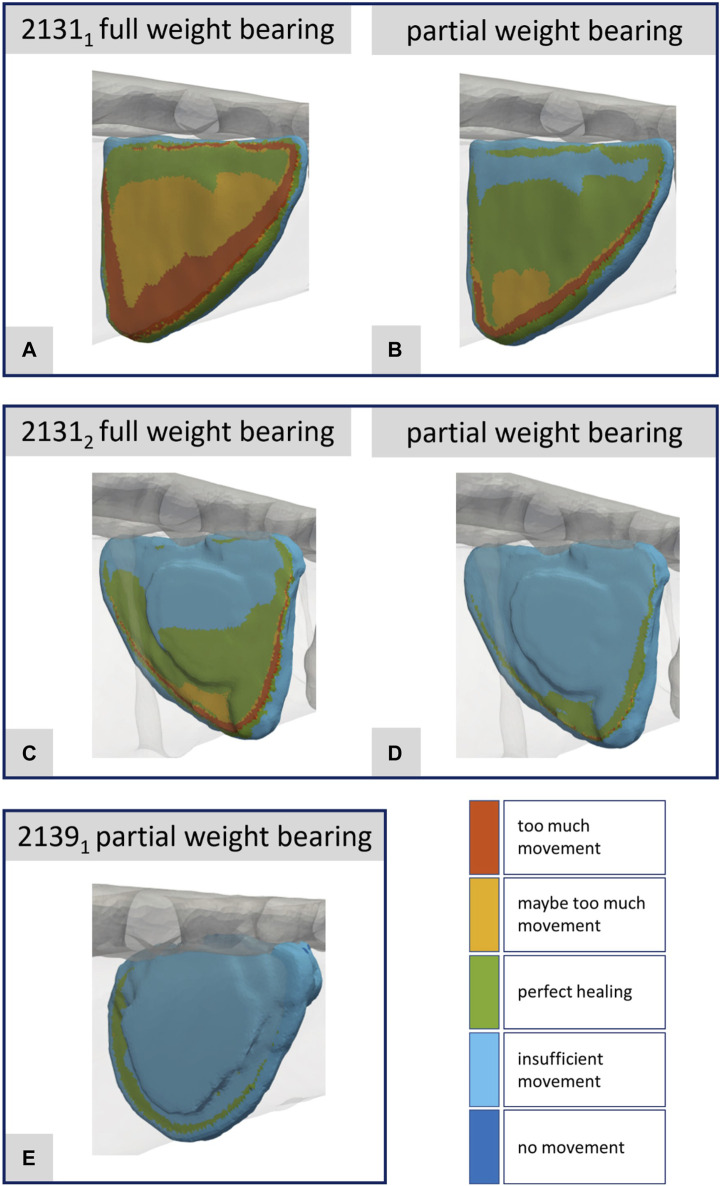
Illustration of the strain-based healing window for all fractures at the time point of the maximal axial force during a normal step forward **(A–E)**. Corresponds to Figures 8A–E.

Table 5 shows additional statistical values for the curves in Figure 10 and enables an evaluation of hypothesis 3. The data provided includes Euclidean distances, MSE, and Pearson correlation coefficients for all experiments. For specimen 2131_1_ under full weight bearing, the Euclidean distance is 0.05785 with an MSE of 3.38e-05 and a correlation coefficient of 0.8401, indicating a substantial positive correlation between the experimental and simulation data. The performance under partial weight bearing shows improved correlation at 0.8726 with reduced Euclidean distance and MSE, suggesting greater accuracy in the simulations under these conditions. Specimen 2131_2_ mirrors this trend, with even higher correlations under partial weight bearing, particularly a correlation coefficient of 0.9495, signifying a very close match between the simulated and experimental data. Specimen 2139_1_, tested only under partial weight bearing, shows an outstanding correlation of 0.9813, the highest among the samples, along with the lowest Euclidean distance and MSE values. These metrics collectively indicate a strong alignment between the simulated and experimental curves, especially under partial weight bearing, thus supporting hypothesis 3, which posits that the simulation model accurately predicts the behavior of the specimens under varying load conditions.

**TABLE 5:** Results for the comparison of the virtual IFM curves from the simulations and the mean IFM curves from the experiments. The table shows the Euclidean distance and the MSE as distance measure between the curves of every experiment, illustrated in Figure 9, and the correlation coefficient from a Pearson test of the data from the curves.

Specimen	Loading	Euclidean distance	MSE	Correlation coefficient
2131_1_	Full weight bearing	0.05785	3.38e-05	0.8401
	Partial weight bearing	0.01800	3.27e-06	0.8726
2131_2_	Full weight bearing	0.05898	3.51e-05	0.8727
	Partial weight bearing	0.01202	1.46e-06	0.9495
2139_1_	Partial weight bearing	0.00408	1.68e-07	0.9813

Figures 10A–E presents the corresponding data for the fracture gap with regard to the strain-based healing window as originally defined by Claes and Heigele (1999) and later by Shefelbine et al. (2005). The color-coded representation demarcates the different fracture regions: areas with too much movement or strain (red), areas likely experiencing too much movement or strain (yellow), and regions identified as beneficial for healing (green). One can clearly see the difference between full and partial weight bearing. For specimen 2131_1_ in particular, large areas change from too much movement to areas favorable for healing when the load input is reduced from full to partial weight bearing. This innovative fracture analysis approach was first showcased in Orth et al. (2023) drawing from a real clinical case and building upon concepts introduced by Braun et al. (2021).

To investigate hypothesis 4, Table 6 presents the evaluation results comparing 3D simulations, reduced to strain energy density within the fracture gap, to 1D relative strain signals from the experiments using the AO Fracture Monitor. For specimen 2131_1_, under full weight bearing, 90th PRC had a Euclidean distance of 0.0274 and an *R* ^2^ of 0.6716, with a correlation coefficient of 0.8204, suggesting a moderate to strong linear relationship as indicated by the regression function. The mean and IQR show similar levels of correlation. The same applies to the quantity’s maximum, median and sum, so that these have been omitted for the sake of clarity. Under partial weight bearing, the 90th PRC slightly increased to 0.0276 with a small reduction in correlation, whereas the mean and IQR values had minimal change. Specimen 2131_2_ showed a better fit in the full weight bearing condition with a PRC Euclidean distance of 0.0241 and a higher *R* ^2^ of 0.7456, and an even stronger correlation coefficient of 0.8635. The regression model for PRC indicates a strong predictive relationship. The mean and IQR also displayed strong correlations, with the IQR yielding the highest *R* ^2^ of 0.7585 under full weight bearing. Partial weight bearing conditions for this specimen also showed strong correlations, with *R* ^2^ values exceeding 0.7598 across statistical values.

**TABLE 6:** Results for the evaluation of the 3D simulations reduced to the strain energy density inside the fracture gap and the 1D relative strain signal from the AO Fracture Monitor from the corresponding experiments.

Specimen	Loading	Statistical value	Euclidean distance	MSE	*R* ^2^	Correlation coefficient	Linear regression
2131_1_	Full weight bearing	90th PRC	0.0274	1.203e-06	0.6716	0.8204	4.8802e-05 * x—0.00086
		MEAN	0.0102	1.191e-06	0.6728	0.8202	1.8358e-05 * x—0.00032
		IQR	0.1030	8.408e-06	0.6716	0.8195	1.8399e-05 * x—0.00025
	Partial weight bearing	90th PRC	0.0276	8.608e-06	0.6654	0.8157	8.7492e-05 * x—0.00141
		MEAN	0.0104	1.218e-06	0.6654	0.8157	3.2921e-05 * x—0.00052
		IQR	0.0104	1.215e-06	0.6683	0.8175	3.3096e-05 * x—0.00046
2131_2_	Full weight bearing	90th PRC	0.0241	6.543e-06	0.7456	0.8635	1.1727e-04 * x—0.00229
		MEAN	0.0090	9.184e-07	0.7478	0.8648	4.4191e-05 * x—0.00086
		IQR	0.0089	8.849e-07	0.7585	0.8709	4.4644e-05 * x—0.00082
	Partial weight bearing	90th PRC	0.0235	6.185e-06	0.7598	0.8715	1.6627e-04 * x—0.00213
		MEAN	0.0088	8.746e-07	0.7598	0.8717	6.2574e-05 * x—0.00079
		IQR	0.0088	8.754e-07	0.7611	0.8724	6.2822e-05 * x—0.00073
2139_1_	Partial weight bearing	90th PRC	0.0199	4.464e-06	0.8264	0.9091	1.3691e-04 * x—0.00147
		MEAN	0.0075	6.289e-07	0.8273	0.9096	5.1543e-05 * x—0.00055
		IQR	0.0074	6.193e-07	0.8310	0.9116	5.1818e-05 * x −0.000488

For specimen 2139_1_, only partial weight bearing data is available, which shows the strongest correlations among the three specimens. The PRC Euclidean distance is 0.0199 with an *R* ^2^ of 0.8264 and a correlation coefficient of 0.9091. The mean and IQR similarly show strong correlations, with *R* ^2^ values above 0.8273 and tightly clustered regression coefficients. These results suggest that the strain energy density derived from 3D simulations correlates well with the 1D experimental strain signals, and the linear regression models provide a good predictive relationship between the simulated and experimental data.

## 4 Discussion

The present study confirmed a significant correlation between the surface strain data of the implant and the IFM as both were derived from the same experiments evaluated via DIC (hypothesis 1). It also established that the 1D measurements captured by the AO Fracture Monitor could predict the IFM measured by DIC, thus linking the implant loading to the behavior of the fracture gap (hypothesis 2). Additionally, the study showed that the simulation results could be reliably evaluated using the experimental DIC data for IFM, especially under partial weight bearing conditions (hypothesis 3). Finally, a strong connection was found between the AO Fracture Monitor’s signals and the simulated IFM, which enabled a transition from 1D to 3D understanding via the strain energy density within the bone-implant system, with linear regression models providing a strong predictive relationship (hypothesis 4). These results, in turn, may be used for clinical application analyses of the AO Fracture Monitor in translational studies.

Both the experiments performed and the simulations based on them are subject to various limitations and few simplifications made. One simplification that had to be made is the use of knee forces from the Orthoload database. These represent the data of the selected patient (k8l) and are only a simplifying assumption as knee forces for the bone donors. In addition, the body weight of the donors was not known, so the body weight of the patient selected from the Orthoload database was adopted here as a simplification. In retrospect, this might have been chosen too high for sample 2139_1_ and could be the reason for the failure of the osteoporotic bone under high weight bearing conditions. Another difficulty that always arises in this type of experiment is the clamping of the specimens. Since bones as a biological and a natural grown structure have a relatively complex geometry compared to standard industrial specimens, a good clamping and alignment of the specimens is challenging. This potentially leads to minor errors in both axial alignment (*z*-axis) and in maintaining the x- and *y*-axes of the clamped bones. The corresponding bone areas are marked in the simulation and the boundary conditions are set there in analogy to the real test execution. Inaccuracies may occur due to the possible slightly offset of the angles caused by the alignment of the specimens and the perfectly aligned simulation models.

Another limitation is the load input to the bone-implant system from the clamping itself and the associated machine setup that occurs as a type of preload or bias and is reflected in the AO Fracture Monitor data because the AO Fracture Monitor cannot technically perform a calibration step after the specimens are installed. The manual calibration step of the AO Fracture Monitor was always done before the start of the particular testing protocols after the installation of the specimens and the alignment and calibration of the camera system. If it were technically possible to combine the calibration step of the AO Fracture Monitor and its data acquisition with the triggering of the testing device, the significance of the data and its subsequent use could be increased even further.

Within the evaluation of the DIC and the software used for this purpose, averaging processes take place that cannot be fully represented in the simulation evaluation. The goal to compare the results of the simulations with the results of the experiments as good as possible can be influenced by using analysis software and the processes running in it. The biomechanical FE simulations were limited to only one step and do not represent all eight steps used as input data in the testing device. This restriction was made because the amount of data for one step in the output database (ODB) files from Abaqus is already up to 100 Gigabyte, making the evaluation time and memory intensive. When simulating the entire input data, the ODB files then reach a size of 800 Gigabyte per specimen and load case, which exceeds the available computer capacity for the evaluation process.

We are aware that the conclusions are drawn from only a small number of experiments, and more experiments from a larger number of donors will have to be provided to further analyze and strengthen the results and associations. Nevertheless, the outcomes of this study suggest that patient-specific simulations in conjunction with the AO Fracture Monitor measurements provide a viable method for assessing the local mechanics within a fracture gap, which is pivotal for understanding mechanotransduction. This methodology can enrich clinical outcomes by enabling personalized healing strategies, refining prognostic accuracy in trauma trials, and offering a sophisticated approach for early detection and intervention in cases of healing complications. Moreover, it has the potential to guide the enhancement of the design of trauma implants, thus improving overall patient care in orthopedic trauma surgery.

## Data Availability

The original contributions presented in the study are included in the article/supplementary material, further inquiries can be directed to the corresponding author.
